# A workflow to identify novel proteins based on the direct mapping of peptide-spectrum-matches to genomic locations

**DOI:** 10.1186/s12859-021-04159-8

**Published:** 2021-05-26

**Authors:** John Anders, Hannes Petruschke, Nico Jehmlich, Sven-Bastiaan Haange, Martin von Bergen, Peter F Stadler

**Affiliations:** 1 Bioinformatics Group, Department of Computer Science, and Interdisciplinary Center for Bioinformatics, Universität Leipzig, Härtelstraße 16–18, 04107 Leipzig, Germany; 2 Institute of Biochemistry, Faculty of Life Sciences, University of Leipzig, Talstraße 33, 04103 Leipzig, Germany; 3 Department of Molecular Systems Biology, Helmholtz Centre for Environmental Research - UFZ, Permoserstrasse 15, 04318 Leipzig, Germany; 4 German Centre for Integrative Biodiversity Research (iDiv) Halle-Jena-Leipzig and Competence Center for Scalable Data Services and Solutions Dresden-Leipzig and Leipzig Research Center for Civilization Diseases, University Leipzig, 04107 Leipzig, Germany; 5 Max Planck Institute for Mathematics in the Sciences, Inselstraße 22, 04103 Leipzig, Germany; 6 Department of Theoretical Chemistry, University of Vienna, Währinger Straße 17, 1090 Vienna, Austria; 7 Faculdad de Ciencias, Universidad Nacional de Colombia, Sede Bogotá, Ciudad Universitaria, Bogotá, DC, 111321 Colombia; 8 Santa Fe Institute, 1399 Hyde Park Rd., Santa Fe, NM87501 USA

**Keywords:** Small proteins, Metaproteogenomics, Peptide-spectrum matches, Microbial communitities

## Abstract

**Background:**

Small Proteins have received increasing attention in recent years. They have in particular been implicated as signals contributing to the coordination of bacterial communities. In genome annotations they are often missing or hidden among large numbers of hypothetical proteins because genome annotation pipelines often exclude short open reading frames or over-predict hypothetical proteins based on simple models. The validation of novel proteins, and in particular of small proteins (sProteins), therefore requires additional evidence. Proteogenomics is considered the gold standard for this purpose. It extends beyond established annotations and includes all possible open reading frames (ORFs) as potential sources of peptides, thus allowing the discovery of novel, unannotated proteins. Typically this results in large numbers of putative novel small proteins fraught with large fractions of false-positive predictions.

**Results:**

We observe that number and quality of the peptide-spectrum matches (PSMs) that map to a candidate ORF can be highly informative for the purpose of distinguishing proteins from spurious ORF annotations. We report here on a workflow that aggregates PSM quality information and local context into simple descriptors and reliably separates likely proteins from the large pool of false-positive, i.e., most likely untranslated ORFs. We investigated the artificial gut microbiome model SIHUMIx, comprising eight different species, for which we validate 5114 proteins that have previously been annotated only as hypothetical ORFs. In addition, we identified 37 non-annotated protein candidates for which we found evidence at the proteomic and transcriptomic level. Half (19) of these candidates have close functional homologs in other species. Another 12 candidates have homologs designated as hypothetical proteins in other species. The remaining six candidates are short (< 100 AA) and are most likely *bona fide* novel proteins.

**Conclusions:**

The aggregation of PSM quality information for predicted ORFs provides a robust and efficient method to identify novel proteins in proteomics data. The workflow is in particular capable of identifying small proteins and frameshift variants. Since PSMs are explicitly mapped to genomic locations, it furthermore facilitates the integration of transcriptomics data and other sources of genome-level information.

**Supplementary Information:**

The online version contains supplementary material available at 10.1186/s12859-021-04159-8.

## Background

Small proteins (sProteins) with a size below 100 amino acids have received increasing attention particularly in prokaryotes. Recent studies have revealed indispensable biological functions of some sProteins. CydX (37 AA), for instance, regulates the activity of cytochrome oxidase and thus ATP production in *E. coli* [1], and SgrT (43 AA) is an inhibitor of the EIICBGlc glucose transporter regulating glucose uptake [2]. Systematic surveys have consistently identified large numbers of sProteins in prokaryotes, see e.g. [3, 4], clarifying it has become clear that sProteins are not rare peculiarities. The human gut microbiome, for instance, features thousands of sProteins, many of which are predicted to function in cell-cell communication [5]. Nevertheless, the available information has remained comparatively sparse due to the technical difficulties associated with their detection and identification using both computational and experimental methods.

The annotation of newly sequenced genomes is primarily based on homology, making use of already existing gene annotations from related species. By definition, this approach is limited to homologs of genes that already been described already in at least one species. The method is also limited by incorrect entries in protein databases. Complementarily, putative coding sequences can be recognized with the help of Markov models that classify open reading frames (ORFs). To obtain a reliable signal, usually a minimum length of 100 codons is required in genome annotation [6]. These methods become unreliable for shorter ORFs, including those compiled in the BactPepDB [7], which surveys all available complete prokaryotic genomes for peptides with a length between 10 and 80 amino acids. Comparative approaches, in particular methods such as RNAcode [8] that evaluate sequence alignments rather than single sequences, can reliably recognize even very short coding sequences. They lose their power, however, if only few genomes within a suitable genetic distance are available. To-date, the computational prediction of sProteins is thus by no means an easy routine task. Ribosome profiling [9] also provides information on translated regions and thus constitutes an alternative manner to identify putative novel proteins.

The gold standard for detecting sProteins is their direct identification in bottom-up proteomics. This technique relies on proteolytically cleaved proteins and subsequent analysis by LC-MS/MS [10]. Classic bottom-up proteomics protocols, however, tend to identify few sProteins since the small size implies that sProteins often yield only a single proteotypic peptide [11–13]. This issue is aggravated by the fact that peptide identification itself depends on underlying databases of predicted polypeptides. Tools such Mascot [14], comet [15], MS-GF+ [16] and many others, therefore cannot identify peptides that are not in the set of protein annotations provided a priori. A peptide identified in this manner is referred to as peptide-spectrum match (PSM).

Proteogenomics approaches typically make use of a conceptual translation of the genome into all six reading frames as the basis for peptide identification. This results in much larger 6frame databases and thus a (moderate) reduction of sensitivity, but completely avoids all annotation-related biases [17–19]. With a focus on sProteins, it is also possible to extend annotations with additional predictions of (short) ORFs with high coding potential [20]. Already two decades ago expressed sequence tag (EST) data were used to predict novel isoforms to allow the identification of proteins arising from splice variants [21]. More recently, the same idea has been used with hypothetical splice variants to identify missense SNPs, short indels, chimeric proteins, and intron retention [18, 19]. Metaproteomics [22], i.e., the application of proteogenomics to entire communities, incurs an additional layer of complexity for data analysis due to the need of to disentangle different, but often closely related species [23, 24].

The focus of this study was the discovery of novel, unannotated proteins, in particular those that have not been flagged as likely candidates by homology-based genome annotation. This problem is more difficult than simply verifying an annotated protein candidate as the overlooked cases are often short, have no or only poorly described homologs in other species, harbor unusual features such as frameshifts, or overlap incorrect annotations. As a consequence, the sensitivity needs to be increased, which necessarily leads to a rapidly growing number of false positive predictions. The estimation of protein-level false discovery rates (FDR) for proteomics data has received extensive attention in the last decade, see e.g.  [25–29]. FDR estimates, however, refer rates estimated across an entire genome. The already annotated proteins are thus heavily biased towards true positives by independent information, and the bulk of the false positives are concentrated in the so-far unannotated genomic regions [30]. Nevertheless, FDR estimates are a useful tool to determine cutoff-values also helpfull in the context of extending existing annotation. Here we describe a workflow to prioritize unannotated candidate proteins based on aggregated quality measures of PSMs mapping to candidate and translational status of overlapping annotation items.

## Results

### Accuracy of identifying candidate proteins

Our goal was to identify *novel* protein candidates with high sensitivity, meaning that we can use the available annotations to exclude already known proteins from further investigation. To identify novel candidates, we start by mapping all PSMs of sufficient quality (see Methods) to the genome and use the genomic map of PSMs to determine candidate proteins using a set of rules based on the quality of the PSMs and the number of PSMs mapping to a putative ORF (see Methods). A candidate within an ORF extends downstream to the closest stop codon, while the upstream end is determined by the first start codon upstream of the upstream-most PSM mapped to the ORF.

**Fig. 1:**
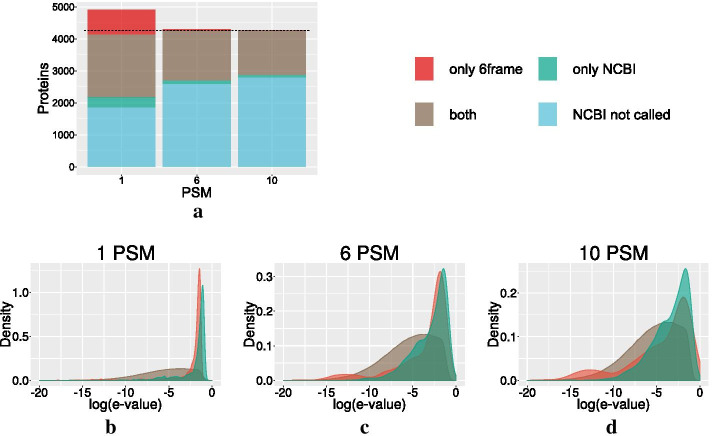
Above: comparison of 6frame and proteome databases for peptide identification using the comet search algorithm. Three barplots are shown for 1, 6, and 10 PSMs required to call a protein. Each bar represents the size of protein sets. Blue shows the cut-off number of proteins which are annotated but not called by the use of any database. Green shows the number of proteins, which are called using the NCBI *E. coli* genome annotation as a database. Grey shows the number of proteins indenpendently detected by both databases. Red shows the number of proteins called by the 6frame data base. The dotted line shows the size of the complete NCBI annotation. Below: The score distributions of the PSMs for different sets of called proteins are shown. The score is given as a common logarithm. Red shows the set of proteins which are only detected by the 6frame database. The green set are the proteins which are only detected by using the NCBI annotation as the search database. Grey is the intersection of proteins that are identified by both databases

In order to determine how well true proteins can be discriminated from false candidates on the basis of properties of mapped PSMs, we use an extensive data set [31] for *E. coli*. The *E. coli* genome is nearly perfectly annotated, meaning unannotated candidates are most likely false positive calls. In addition, we compare candidate calls using a 6frame database with calls based on a database of annotated proteins (proteome). While we expect that the sensitivity of 6frame is reduced compared to proteome, we can use candidates found only with the 6frame but not with the proteome database to estimate the false positive rate.

If only a single PSM is required to identify a protein, we observe in that the majority of the annotated *E. coli* proteins are called using both the 6frame database and annotation-based database. Figure 1a shows that there is nevertheless a noticeable differences between the two databases. The consistency increases rapidly if more—not necessarily distinct—PSMs are required, see also Additional file 1: Figure S1. While recovery is reduced by about 15% and 20%, respectively, the two methods yield nearly identical results when 6 or 10 PSMs are required. In the 6frame-based data, very few unannotated candidates remain. Discrepancies between the databases are related almost exclusively to PSMs with poor scores, Fig. 1b–d. Candidate proteins that are only detected by one of the two database show a strong accumulation of poorly scoring PSMs. Therefore they can be interpreted as mostly false positives.

FDRs estimated using different approaches from the PSM scores consistently indicate that the number of observed non-annotated loci can be explained by the expected number of false positives (see Methods). We therefore expect most of the unannotated loci to be false positives. That does not imply, however, that a small-to-moderate fraction cannot be true positives. We therefore ask whether the distribution of PSM scores, i.e., the confidence with which they are identified from the MS/MS spectra, can be leveraged. First, we observe that most PSMs that are mapped by one but not the other database are of low confidence. Low confidence PSMs, furthermore, are strongly enriched in proteins to which only very few PSMs are mapped, Fig. 1b–d. This matches the observation that false positive PSMs accumulate among unannotated ORFs [30]. It is common practise in proteomics to aggregate statistics of PSM quality values to produce a score describing the confidence of protein identification [26–29]. Our observations suggest that such aggregate scores will also be useful for the purpose of extending existing annotations.

**Fig. 2:**
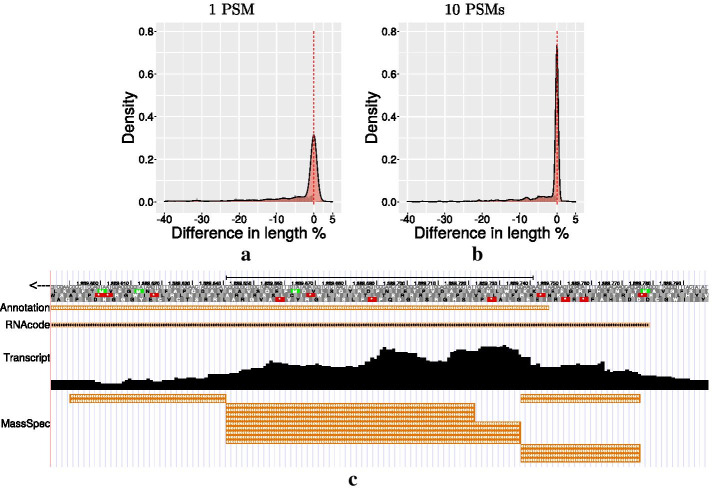
Comparison between the length difference of candidates from 6frame search and the NCBI annotation of *E. coli*, expressed as fraction of the total protein length. A value of $0\%$ (red dotted line) indicates that candidate and annotation are identical. Negative values mean that the candidate is shorter than the annotated protein due to lack of coverage by PSMs towards the N-terminus. Positive values imply that the candidate has a longer N-terminal sequence than that indicated in the annotation. Below, the N-terminus of the fatty acyl-CoA synthetase *FadD* is shown in the UCSC Genome Browser. The first track shows the reading frame of the genome on the negative strand. The second track shows the current annotation. After this the gene annotation is shown as predicted by RNAcode [8]. The next line shows a bar plot representation of the mapped reads per base of the transcriptomic data. At the bottom track the mapped PSMs are depicted. It is misannotated and extends to canonical start. Our extension of this reading-frame is supported by evidence of conserved coding sequences determined by RNAcode

As a second measure of how well we are able replicate the original annotation using a 6frame database, we quantify the differences between start sites predicted with the 6frame database and start sites reported in the original annotation. Their 3’-ends match perfectly as they are determined by the same in-frame stop codons. For most of the annotated candidates, we recover the original length of the annotated protein (dominating peak at 0 in Fig. 2). For a fraction of the data we predict shorter candidates than those in the original annotation, presumably due to a lack of PSM coverage on the N-terminal part of the candidate. In a small number of cases our candidates begin upstream of the given annotation. This concerns 24 proteins with 6 PSMs. Fig. 2 shows one example, the fatty acyl-CoA synthetase *FadD*. Here, PSM evidence clearly shows that the true start codon is located upstream of the annotated coding sequence (CDS). Similar arguments can be made for 4 of the 24 cases with extended N-termini, the full list can be found on the result web page. That is set up as part of the supplementary material. Indicating that despite the outstanding quality of the annotation of the *E. coli* K12 reference genome, it is still not perfect and proteogenomics data are able to correct some of the remaining inaccuracies.

This observation prompted us to also inspect in detail the 11 “false positives” that are supported by 10 or more PSMs. It turns out that two of them corresponded to two parts of the formate dehydrogenase O subunit alpha, which our pipeline did not recognize due to a (presumably erroneous) stop codon in the genomic sequence. Two candidates are the two parts of the peptide chain release factor RF2, which has long been known to contain an obligatory frameshift [32]. Its peptides thus appear in two distinct predicted ORFs, neither of which completely matches the annotation. Several mRNAs in *E. coli* are known to produce minor variants that include a frameshift [33]. Two additional candidates are an IS5 transposase, for which frameshift has also been reported [34], and the transcriptional regulator GlpR, which, according to the UniProt annotation also harbors a frameshift.

This leaves only 5 ORFs as likely false positives. Surprisingly, these candidates are well distinguished by the distribution of PSM scores: while the frameshift proteins harbor mostly well-scoring PSMs, the remaining, likely false positives are matched only by PSMs with poor scores. This observation further supports the idea to aggregate PSM quality statistics as a useful tool for protein prediction. It also advises against simply adding $-\log (\text {e-value})$ scores in order to predict proteins and instead suggests to use the best observed value, in line with the proposal of the PCM score (Best scoring PSM per peptide charge modification combination) in [29].

Increasing sensitivity through specifying 6 PSMs per candidate moderately increases the number of candidate proteins to 29. Using the number of candidates predicted with a 6frame proteogenomics approach, that do not match the annotation (or are not called using a proteome database) shows that the FDR quickly drops with the number of PSMs that are required to call a candidate, Addition file 1: Figure S2.

The proof reader suggested to move the section below to move to the discussion

Our analysis of the *E. coli* data suggests that a coverage of 6–10 PSMs is sufficient to identify likely candidate proteins. Notably, these PSMs may correspond to the same peptide. Combining this minimum coverage with a simple aggregate score derived from the $-\log (\text {e-value})$ data of the individual PSMs readily allows the distinguish the recognizable proteins in *E. coli* form those that our detailed analysis classified as false positives. The detailed inspection of the data also suggested utilizing the average of the $-\log (\text {e-value})$ score of the three best-scoring PSMs as a more robust measure than simply opting for the optimal $-\log (\text {e-value})$. It is unlikely that the *E. coli* genome harbors many undiscovered candidates. We therefore analyse a larger, much less well annotated data set next.

### Metaproteogenomics of SIHUMIx

The proteomics data for SIHUMIx was analyzed using a combined 6frame database for the eight species. In order to verify that this approach can properly separate the spectra from the different species we determined the number of PSMs mapped to more than one species, shown in Table 1. More than 95% of the PSMs are unique, and thus can be unambiguously assigned to one of the species of the consortium. The majority of the remaining PSMs matches only two positions on the metagenome (multiplicity$=2$) either in the same or in two distinct species. PSMs with high levels of ambiguity are exceedingly rare, reflecting the fact that members of the SIHUMIx are phylogenetically quite distant from each other.

**Table 1:** Ambiguous mapping of PSMs in the SIHUMIx dataset with proteome and 6frame databases

Multiplicity	Proteome (%)	6frame (%)
1	0.9582	0.9599
2	0.0288	0.0271
3	0.0056	0.0051
4	0.0052	0.0051
5	0.0009	0.0014
6	0.0006	0.0006

Multiplicity refers to the number of distinct loci in the metagenome to which a PSM maps

**Fig. 3:**
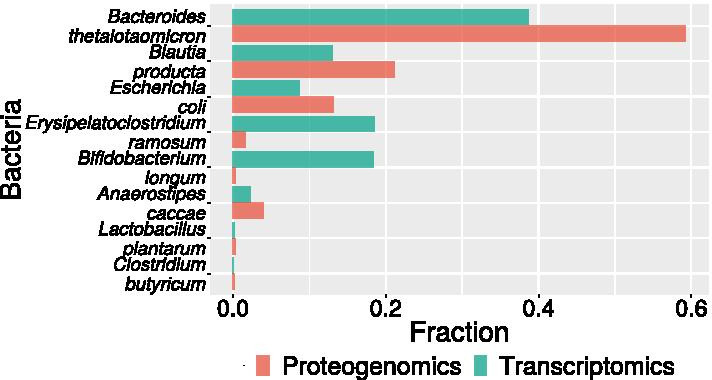
Species composition of SIHUMIx from proteogenomic (red) and transcriptome data (blue). The relative frequency is given as the number of PSMs per species divided by the total number of PSMs for the in the proteogenomic dataset, respectively the number of reads for transcriptome dataset. Both the mapped numbers of reads and the number of PSMs were normalized to complete genomic size and proteogenomic (6frame DB) size respectively

For this model system we also analysed extensive RNA-seq data as a means of supporting proteogenomics-based predictions. It is not unexpected that there is only moderate agreement between protein and RNA abundances in Fig. 3, since RNA/protein ratios are known to vary considerably between organisms [35].

**Fig. 4:**
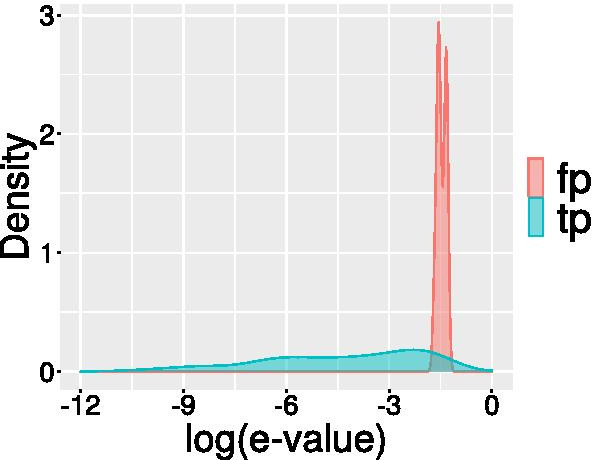
Score distributions of the PSMs mapping to the unannotated candidate proteins in *E. coli*. Each candidate was manually inpected by checking its genomic context and transcriptomic data. Those identified as likely true positives harbour PSMs with excellent scores, while those identified as likely false positives harbour only low-scoring PSMs

The rate of detection of known and hypothetical proteins in the eight SIHUMIx species, as expected, correlates with the relative abundance in the mixture, see Table 2. There is near perfect congruence between 6frame and proteome databases, see Additional file 1: Table S1.

**Table 2:** Summary of the number of proteins detected with at least 10 or 6 PSMs in the SIHUMIx proteogenomic dataset, using the 6frame translation of the genomes

Species	Nov	Hyp	Known	%
At least 10 PSMs per candidate				
*B. theta.*	37	1975	248	45.9
*B. producta*	52	1138	132	23.2
*E. coli*	26	150	988	26.8
*E. ramosum*	10	355	53	13.7
*B. longum*	16	128	0	7.4
*A. caccae*	17	549	100	19.3
*L. plantarum*	31	83	28	3.7
*C. butyricum*	14	135	32	4.1

Species	Nov	Hyp	Known	%
A least 6 PSMs per candidate				
*B. theta.*	72	2118	256	49.0
*B. producta*	103	1289	143	26.1
*E. coli*	65	182	1127	30.9
*E. ramosum*	30	431	65	16.7
*B. longum*	42	170	0	9.8
*A. caccae*	39	632	119	22.3
*L. plantarum*	48	116	36	5.1
*C. butyricum*	26	176	39	5.3

Novel (nov) proteins are not contained within annotation, hypothetical (hyp) proteins are annotated but tagged with low confidence (see “Methods” section for details), known refer to all proteins for which higher levels of confidence are associated with the available annotation. The eight species are ordered by decreasing abundance. The last column gives the fraction of the annotated proteins that were detected

The distributions of known and hypothetical proteins differs dramatically across the eight SIHUMIx species. In most species, the majority of proteins are annotated as hypothetical based on the quality of evidence. Since the confidence levels are unlikely to be truly consistent between species due to differences in the efforts that have been expended for their annotation, this figure should however be interpreted with caution. The proportion of known and hypothetical proteins, however, at least reflect qualitative trends.

### Novel proteins in SIHUMIx

We discovered a total of 419 unannotated protein candidates supported by at least 6 PSMs in SIHUMIx. Since these initial candidates also include all those predictions that overlap annotated proteins in a different reading frame, we expected a priori that most of them would be false positives. While it is manageable to manually evaluate a few hundred candidate proteins in a data set of particular interest, this is not practical for routine applications and thus requires computational support. In order to better understand this candidate set we systematically gathered all information on the protein candidates within it that were readily accessible by computational means. This leads to a natural workflow for prioritizing and validating.

**Fig. 5:**
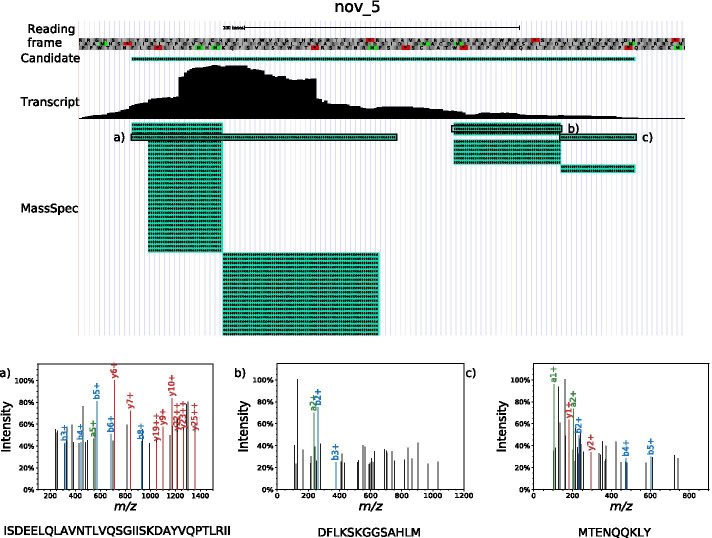
Protein candidate 5 in *Blautia producta*, for which no homologs can be found. The top of the figure shows a view in the UCSC Genome Browser. The first track show the reading frame of the genome on the negative strand. The second track is the protein candidate 5’s predicted ORF. The next track shows a bar plot representation the reads per base mapping to protein candidate 5 from the transcriptomic data. At the bottom a list of the mapped PSMs can be found. A truncated list of the 168 mapped PSMs is displayed. Below three mass spectra with top confidence PSMs and the corresponding peptides are shown

**Fig. 6:**
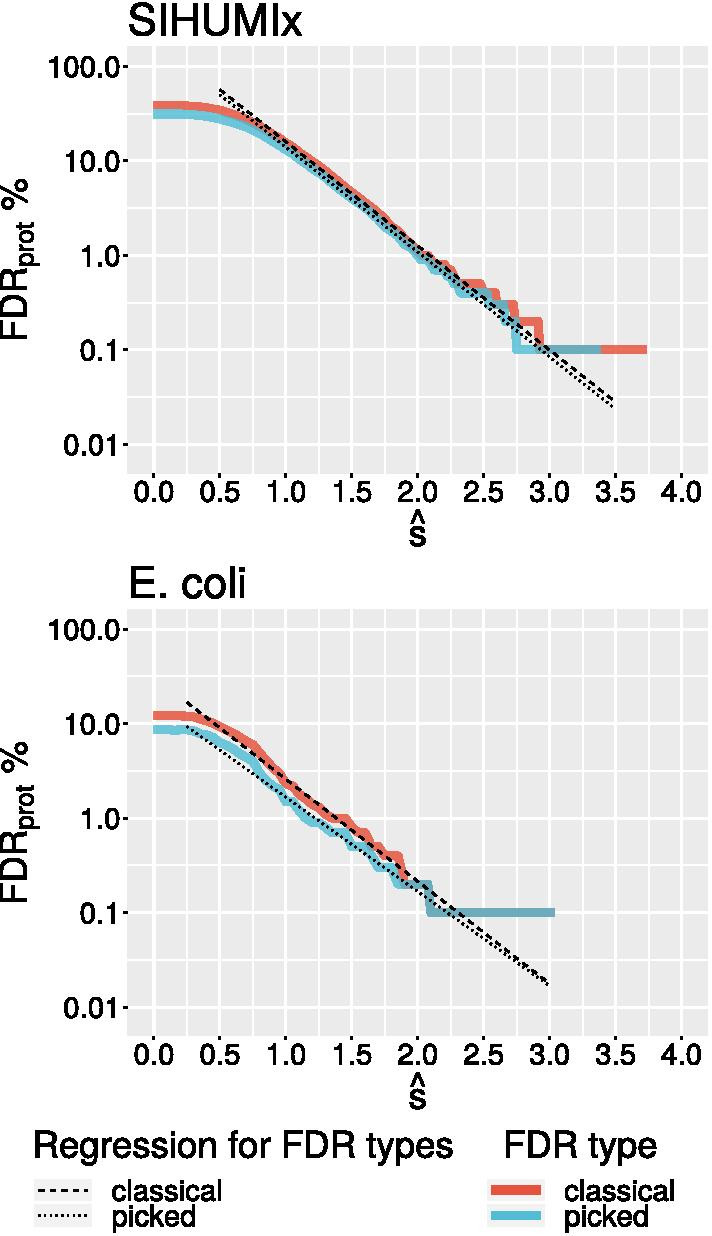
FDR$_{prot}$ computed from $\hat{s}$ values for proteins with at least 6 mapped PSMs in the SIHUMIx data set (see Methods). Two strategies are used to compute the FDR$_{prot}$, the “classical” and the “picked” strategy (see [29] and the “Methods” section). The regression lines extrapolating to large value of $\hat{s}$, for which the decoy database produced no hits, is computed from the $\hat{s}$-score interval [1.0, 2.5] for SIHUMIx and [0.75, 2] for $0.75-2$ *E. coli*. The cutoff-value $\hat{s}=3.5$ for SIHUMIx corresponds to a *q*-value of 0.03. For the much smaller *E. coli* data set, the same *q*-value is is obtained for $\hat{s}\approx 2.7$

**Fig. 7:**
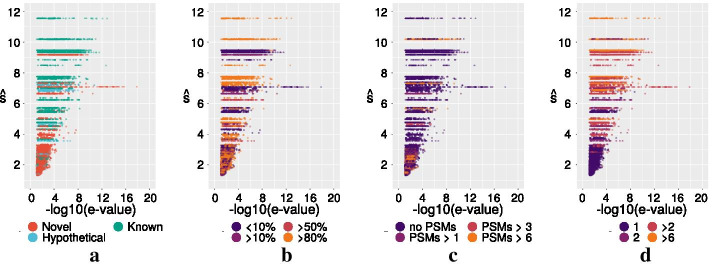
Distribution of $-\log (\text {e-value})$ for the PSMs contributing to each candidate. All candidates with at least 6 mapped PSMs are shown. Each dot represents a single PSM. On the Y-Axis the median of the top three PSMs $\hat{s}$ is plotted. Thus the observed horizontal lines of the dots show all PSMs belonging to one candidate. For all figures we provided interactive plots which can be found under [36]. There each individual PSM/dot can be inspected and mapped to its corresponding candidate. **a** Candidates with known functionally annotated homologs (green), homologous hypothetical proteins (blue), and no homologs/ novel (red) in the non redundant NCBI protein database are distinguished. **b** The same data is colored by RNA expression level.Given in percent of the ORF, which is elevated above the average of the genome. Ranging from low (purple) to high (orange) expression. **c** Whether translation (number of PSMs) was observed in a different ORF of the same or the opposite reading directions, and in **d** color coding refers to number of distinct peptide contributing to a candidate

A homology search against the non redundant NCBI protein database identified 60 of the 419 candidates with extensive similarity to proteins with a functional annotation in related species. These cases are clearly shortcomings in the available annotations of the genomes in SIHUMIx and constitute a positive control for our approach and help to establish criteria, which can be applied to analysis the remaining candidates. We exclude these 60 proteins from further analysis as we are primarily interested in those candidates proteins that cannot be found using traditional homology-based methods. In addition to these homologs of known proteins, we identified another 47 of the 419 candidates that are homologs of hypothetical proteins.

To establish criteria for prioritization and validation, we first considered the distribution of the e-values of the PSMs that contribute to each candidate protein. The data in Fig. 4 already strongly suggest that this is a reliable predictor. We use the average $\hat{s}$ of the scores $s :=-\log (\text {e-value})$ for the three best PSMs as an aggregate descriptor. Figure 7 summarizes all candidates with at least 6 supporting PSMs. Figure 6 shows the protein-level FDRs as a function of $\hat{s}$. Almost all candidates with $\hat{s}>3.5$, corresponding to a *q*-value of about 0.03 for the SIHUMIx data set, have homologous known proteins in other species. As an example, the *B. producta* candidate nov_57 is shown in Additional file 1: Figure S4. (top). It has a probable length of 72 amino acids and shows a recognizable homology with adenylate kinase of similar length from Listeria.

In total, 47 of the 419 candidates have $\hat{s}>3.5$. We first inspect all candidates with more than 10 high scoring PSMs. Interspersed among these known genes are three novel proteins (*B. producta* nov_5, *B. theta.* nov_59 and nov_131). Nov_5 is clearly a complete protein, while nov_59 and nov_131 may be associated with frameshifts and constitute only parts of a protein. The most prominent candidate, *B. producta* nov_5 is shown in Fig. 5, lower panel. It has a likely length of 62 amino acids, judging from both the observed PSMs and the transcriptome data. Most but not all of these high-confidence candidates show evidence of transcription. Low RNA levels do not necessarily imply that the predicted protein is a false positive. In fact detection limits for RNA and protein may be vastly different. The typically much longer half-life of proteins may also contribute to explaining the presence of protein with low or undetectable RNA levels.

In five cases (*B. producta* nov_1, *E. coli* nov_8, *B. theta.* nov_34, *B. theta.* nov_131, *B. theta.* nov_61) there are also annotated proteins in the same reading direction. Owing to our definition of the candidates, which extends to the nearest in-frame stop- and the nearest in-frame start-codon, this kind of overlap indicates either an annotation error or a frameshift. Inspection shows that for *B. producta* nov_1 the available annotation of a TetR family transcriptional regulator extends across the stop codon. The remaining signals likely pertain to frameshifts. For *B. producta* nov_126 there is only weak evidence for translation of the annotated gene on the opposite strand, and convincing evidence for translation of a Cna B-type domain-containing protein corresponding to nov_126 that has been left unannotated.

Only three candidates with 6-9 PSMs have $\hat{s}\ge 3.5$: *B. producta* nov_216, an IS66 family transposase, nov_307 a hypothetical protein without functional annotation, and *E. coli* nov_302, the frameshift fragment of peptide chain release factor RF2 already discussed above.

The analysis of the remaining candidates with $\hat{s}<3.5$ is much less straightforward. Although the overwhelming majority of them shows no homology to a known or hypothetical protein, this set contains at least a small number of proteins with known homologs with convincing proteomics evidence: *B. producta* nov_174 $\hat{s}=3.3$, *B. producta* nov_215 $\hat{s}=2.9$, *B. theta.* nov_180 $\hat{s}=2.8$, and possibly *E. coli* nov_122 $\hat{s}=2.3$. Some others, such as *B. producta* nov_84 $\hat{s}=3.0$ and nov_28 $\hat{s}=2.4$, however, are almost certainly false positives. A few curious cases, such as *E. coli* nov_123, $\hat{s}=2.1$, are indicative of incorrect stop-codons or read-through; here the candidate sequence matches a GntR family protein from related species whose sequences extend beyond the stop codon of the annotated *E. coli* GntR gene immediately upstream of nov_123.

Protein expression of the opposite strand is a good indication that a candidate is a false positive: while overlapping ORFs are not uncommon in bacteria, long overlaps of coding regions are very rare [37, 38]. There are, however, a handful of exceptions. As already mentioned above, *B. producta* nov_126 is much more plausible than the potentially expressed ORF on the opposite strand. A few additional cases are supported by many good PSMs mapping to two or more distinct peptides. The best example in our data is *L. plantarum* nov_19, $\hat{s}=3.28$, which would be an interesting candidate for further study.

For moderate values of $\hat{s}<3.5$, therefore, we need additional criteria to distinguish between *bona fide* protein detections, novel fragments of already known proteins that should prompt an update of known protein, and false positives. We therefore inspected additional descriptors. First, we considered the number of distinct peptides corresponding to the PSMs belonging to a given candidate. Supporting the use of $\hat{s}$ as a valuable indicator, we found that with few exceptions, the candidates with large $\hat{s}$ values have multiple peptides, while for small $\hat{s}$, most candidates are supported only by a single peptide. The few notable exceptions (nov_174, nov_215, nov_180) with more than 3 distinct peptides have already been identified above as proteins with known homologs.

### Workflow for identifying and prioritizing candidate proteins

**Fig. 8:**
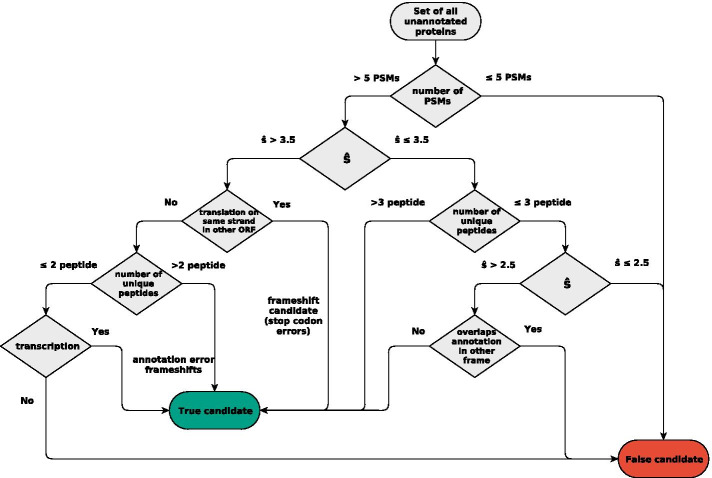
Rules to prioritize candidate proteins for further investigation. A candidate is classified as transcribed if more than 70% of its length is above the median RNA expression level of the organism. Annotated genes which overlap a candidate are classified as translated if they are identified by more than 6 unique PSMs. The $\hat{s}$ cut-offs values can be adjusted according estimates of genome-wide protein-level FDRs, see text

The detailed evaluation of both the *E. coli* and the SIHUMIx metaproteomics data reported above informs the workflow for the identification of novel proteins shown in Fig. 8. It primarily relies of the number of PSMs mapped to an ORF and the distribution of their e-values, irrespective of whether or not there are multiple distinct peptides. The initial decision is based on the number of PSMs, followed by a cut-off on the average score of the three best PSMs. Together the two values ensure reproducibility of good matches in the data set. For values of $\hat{s}\ge 3.5$, unlikely candidates are only those without distinct peptide matches and no evidence for transcription. For values $2.5\le \hat{s}<3.5$ multiple distinct peptides may rescue an initial negative decision. Here, transcriptomics data are not decisive, since prokaryotic genomes produce diverse non-coding transcripts [39–41], so that transcription in itself cannot be used as a reliable predictor of translation.

## Discussion

We have shown here that prokaryotic proteins can be identified with high reliability by considering the PSMs that map to their corresponding genomic locations. Using SIHUMIx as an example we found that $\hat{s}$ (the average logarithm of the e-value of the best few PSMs that map to a candidate ORF) is an excellent discriminator between *bona fide* proteins and other false positive signals. In conjunction with the number of PSMs, it is sufficient to identify nearly all of the ORFs in the SIHUMIx data that have functionally annotated homologs in related species and thus are most likely true proteins. In a fine-grained analysis, the number of distinct peptides helps to distinguish likely candidates from background noise in the case of moderate values of $\hat{s}$. Manual inspection also revealed that translation products involving frameshifts can be detected even if the frame-shifted part contains only a single detectable peptide. Somewhat surprisingly, RNA expression data added very little to the task of identifying novel proteins.

The choice of the cut-off values for the minimum number of PSMs and $\hat{s}$ can be grounded more soundly in established statistical procedures by computing FDR$_{\text {prot}}$ curves as a function of these two parameters. For a fixed minimum number of PSMs this allows a translation of $\hat{s}$ into a *q*-value. Empirically, we observed that most of the plausible candidates are found within q-value cut-offs of 0.01 and 0.1. It is important to note, however, that this *q*-value refers to the complete proteome and therefore to the task of annotating proteins *de novo* in a unannotated genome. It does not have a clear quantitative interpretation for the application scenario considered here, namely the *extension* of an a priori given annotation of protein-coding regions. In this setting, the overwhelming majority of true positives correspond to already annotated proteins, which are typically supported by multiple sources of independent information, such as homologous proteins in other species, codon usage patterns, or independent proteomics studies. As a consequence, the false positives are concentrated among the PSMs mapping to unannotated regions. This effect also has been discussed previously [30]. Starting from a good homology based annotation, which is usually generated with publication of genomes, the FDR *when restricted to* must be expected to be magnitude larger than on the whole, genome-wide data set.

The completion of a (good) genome annotation thus is not merely an issue of cut-off values but requires a workflow that (a) limits the candidate set, (b) allows prioritisation of the candidates, and (c) makes it easy to combine proteomics information with other data sources. Our proposal for such a workflow is summarized in Fig. 8. It is designed to efficiently identify previously unannotated candidate proteins. It can also be employed to validate previously annotated proteins using the same decision criteria, since it accurately reproduces the annotation of known proteins from the PSM data and in some cases identify annotation errors such as incorrect start codons. For this latter mode of action, cut-off values for $\hat{s}$ correspond to desired *q*-values, i.e., thresholds for the protein-level false discovery rates. Despite the expected accumulation of false positives in the unannotated part of the genome, we find *empirically* that candidates with scores corresponding to genome-wide *q*-values below 0.1 or 0.01 could be validated as *bona fide* proteins using external information. It is also worth noting that candidates with very few PSMs are exceedingly rare in this range. For $\hat{s}$ scores slightly below this cut-off level, plausible candidates expectedly become rare. Nevertheless, we found several new candidate proteins below this cut-off showing that it is a worthwhile endeavor to inspect them in detail.

The fact that our workflow maps PSMs directly to the genomic sequence is very helpful for this purpose. It enables the visualization of the data in standard genome browsers and thus greatly facilitates the integration with other data sources, in particular transcriptome data and information on sequence conservation. The presentation of the data in a genome browser supports the manual evaluation of protein candidates in their genomic context, because information of overlapping features, including predicted proteins and PSM data mapping to other reading frames is directly accessible.

Candidates identified as (likely) novel proteins can be further characterised computationally. Most importantly, a homology search is likely to identify a large fraction of candidates as homologs of proteins that have been described already in other species. As in the case of the SIHUMIx example, we expect this to leave only a small fraction of novel proteins and homologs that so far have appeared only as “hypothetical proteins”.

The workflow of Fig. 8 provides a robust way to identify novel proteins, including sProteins, from large mass spectrometry data sets. The method is applicable not only to a single species but also to metaproteomics data, provided the species composition of the sample is known. In the artificial gut community SIHUMIx we found 37 non-annotated novel proteins, among them six sProteins. Applications to microbial communities, however, are likely to be limited to the most abundant species, since the probability to identifying a protein depends on its relative abundance in the sample.

## Materials and methods

### Proteomics data sets

For our analysis we used two different tandem mass spectrometry data sets. One is a data set from a single strain *E. coli K-12*, grown under standard conditions (16h growth in LB medium at 37 $^{\circ }$C with shaking). The data set consists of seven experimental replicates and is part [31].

The SIHUMIx datasets are described in detail in [42–44]. They comprise 166 independent measurements, of which 90 used a standard protein preparation protocol and the remaining 76 cover different enrichment protocols to elevate the level of small proteins in solution. Over all data sets roughly 9.2 million spectras where measured. For the ecoli MS/MS-data set searching against both data bases over 400 thousand PSMs where anlaysed with a $FDR_{decoy}$ cut off at 1%. The search of the SIHUMIx data sets against the 6frame data base (the main analysis to find new protein candidates) resulted in over 2,5 million PSMs with a $FDR_{decoy}$ cut off at 1%. Beside different protein enrichment protocols, both Trypsin (145 protocols) and Asp-N (21 protocols) were used as different cleavage enzymes.

### Peptide identification

We used getorf [45] (Version 6.6) to retrieve all open reading frames between two stop codons from the genomic DNA sequence without any length constraints. For each ORF we store its amino acid sequence as well as its genomic start and end coordinates. The reading frame is defined as that start coordinate $k \bmod 3$ in forward direction and $(k \bmod 3) -3$ in negative direction. We then used Comet [15, 46] (Version 2019.01 rev. 4) to search tandem mass spectra against protein sequence databases. Standard search parameters were used from both the 6frame and the annotated protein databases, with the following exceptions: (i) we allowed semi-digestion at the N-terminus to accommodate fragmentation at the start codon, (ii) we conducted a concatenated search against a decoy database, and (iii) we minimised the bin size of the scans, to increase the resolution of the MS/MS spectra.

### Estimation of false discovery rates for PSMs

The $FDR_{decoy}$ is obtained by counting the number of PSMs for a fixed quality score in both the decoy and the target database. As it is assumed that a falsely assigned PSM is equally likely to happen in the decoy and the target data base. The $FDR_{decoy}$ can be estimated as ${\#decoy PSMs}/{\#target PSMs}$. For the estimation we used the e-value as computed by comet. Two alternative approaches to estimate the FDR have been proposed [30, 47]. Both make use of the assumption that false positive PSMs are mapped with equal rate to a translated and non-translated locus. Ignoring the possibility of overlapping proteins in different frames one interprets all *n* PSMs mapping to one of the five incorrect reading frames of an annotated protein as false positives, resulting in an estimated number of (6/5)*n* false positives. Of the *N* PSMs mapping in the correct reading frame, one therefore expects $N- (1/5) n$ to be true positives. We can therefore estimate the false discovery rate as

1 $$\begin{aligned} \mathop {FDR}_{ann} = \frac{6}{5} \frac{n}{N+n} \end{aligned}$$

where $n+N$ is the total number of PSMs mapped to an annotated locus irrespective of the frame.

Alternatively, we make the assumption that the protein annotation, which covers a fraction $\alpha$ of the genome, is complete. All $n'$ PSMs mapped outside this annotation are counted as false positives. This yields the estimate

2 $$\begin{aligned} \mathop {FDR}_{genom} = \frac{1}{1-\alpha } \frac{n'}{N'} \end{aligned}$$

where $N'=N+n$ is the total number of mapped spectra. The prefactor extrapolates the same FDR to the annotated part of the genomes. In order to account for very short ORFs to which no ORFs can be mapped by construction, the factor $\alpha$ can be estimated more accurately by estimating the chance that a randomly drawn PSM from the 6-frame annotation falls into an annotated region. For *E. coli* this yields $\alpha =0.293$. We note that $FDR_{ann}$ is by construction robust against incomplete annotation and also will not change substantially if there are some incorrectly annotated genes. In contrast, $FDR_{genome}$ will only produce good estimates for genomes with reasonably complete annotations [30].

We checked consistency of these two FDR estimates for the *E. coli* data. Among the $N=180059$ mapped PSMs we observed $n=829$ hits to an incorrect reading frames obtain $FDR_{ann}=0.55\%$, i.e., a slight improvement over comet’s internal estimate of $1\%$ from hits in the decoy database. Alternatively, at least in a well-annotated genome such as *E. coli* we may use PSMs mapped to unannotated regions as an estimator. This yields $FDR_{genome}=0.52\%$. We also validated that, as expected [30] the genome-based FDR estimates are proportional to the FDRs estimated for the decoy database (Additional file 1: Figure S3).

### Estimation of false discovery rates for proteins

The most widely used method to predict a $FDR_{prot}$ is to extend the idea of $FDR_{decoy}$ for PSMs to the protein level. To this end, an aggregate score *s* is computed from the quality scores of all PSMs that map to a candidate protein. In the “classical” $FDR_{prot}$ approach, for a fixed cut-off of *s*, the number of decoy proteins *n* and the number of target proteins *N* are counted, which are identified above a specific score [25, 26]. As an improvement the *picked target decoy* method was proposed in [29], which promises to be more robust than the simple $FDR_{prot}$ estimate. In the *picked target decoy* strategy a protein is only counted if its “mirrored” (or otherwise scrambled) counterpart scores worse. Both strategies were used to calculate the $FDR_{prot}$ with a PSM cut-off of 6 for the $\hat{s}$-score, defined as the average of the three best values of $s=-\log (\text {e-value})$ as computed by comet.

The estimates of $FDR_{genome}$ and $FDR_{ann}$ are extended to protein in the same manner. Since in this case the definition of a “false positive” depends only on the genomic position to which it is mapped, there is of course no analog to the “picked” method. This strategy was used to investigate more closely the influence of PSM cut-offs on the false positive rate (Additional file 1: Figure S2). We obtained consistent results from the different estimates.

### Mapping PSMs to the genome

To map PSMs to the genome, we determine its relative position in the ORF or ORFs of the protein or 6frame database. This position is then directly translated to the genomic coordinates using the known genomic coordinates of the ORFs/proteins. A given peptide sequence may map to multiple ORFs/proteins. If this is the case, the multiplicity of the mapping is stored and can be accessed in the genome browser.

### Construction and annotation of candidate proteins

We start from the collection of ORFs for a genome. For each ORF, we determine all PSMs that map within it. The C-terminus of the candidate is determined by the stop codon of the ORF. The N-terminus is the closest canonical start codon before the first mapped PSM, or if no such start codon exists within the ORF, the first position of the ORF.

The candidate proteins are then compared to the protein annotation that is available for the genome in question. A candidate is considered annotated if it overlaps an annotation item in the correct reading frame and reading direction. In each case, we record the difference between the genomic start positions of annotation and candidate.

Protein contained in the available annotations are classified as *known* unless they are tagged with validation levels 1, protein uncertain or 2, protein predicted in UniProt (i.e., lacking evidence from experiment or homology), or carry the annotations frameshifted, internal stop, hypothetical, Putative, or pseudogene. All of these are interpreted as *hypothetical* in Tabel 2.

### Transcriptome data

The transcriptome data were taken from [31] and mapped with segemehl [48] (Version 0.3.4) to an index comprising the eight SIHUMIx species as separate chromosomes. Default parameters were used. Annotation files were generated with samtools (http://www.htslib.org/, Version 1.1). Total expression per species was averaged over all replicates.

### Visualization

We display the data using the UCSC genome browser [49], which make it easy to integrate them with other data, including transcriptome data, available annotations, as well as custom annotations. See below for the data made available with this contribution.

## Supplementary Information

**Additional file 1.** Supplemental Figures andTables.

## Data Availability

The transcriptomics data is available under the bioproject PRJNA655119 [50]. The mass spectrometry proteomics data have been deposited to the ProteomeXchange Consortium via the PRIDE [51] partner repository with the dataset identifier PXD023243. The genomes and corresponding annotations used for the project are all publicly available by The NCBI Assembly database [52] a full list can be found in Additional file 1: Table S2. The following material is available for download from [53]: $\bullet$ SIHUMIx track hub (track hub for the UCSC genome browser) $\bullet$ Result web page (full list of candidates, ecoli annotation errors and interactive plots) $\bullet$ Validation hash map (Maps each annotated protein in SIHUMIx to a validation level as of the time of the publication) All scripts which are used to generated the data for this publication are available under [54].

## Abbreviations

AA: Amino acid
EST: Expressed sequence tag
FDR: False discovery rate
ORF: Open reading frame
PSM: Peptide-spectrum match
