# Supplementary material for: A workflow to identify novel proteins based on the direct mapping of peptide-spectrum-matches to genomic locations
Source: BMC Bioinformatics. 2021 May 26;22:277. doi: 10.1186/s12859-021-04159-8 (PMC8157683; doi:10.1186/s12859-021-04159-8)
Supplement: Supplementary file 1 — Additional file 1. Supplemental Figures andTables. [file 12859_2021_4159_MOESM1_ESM.pdf]

ADDITIONAL FILE 1

SUPPLEMENTAL FIGURES AND TABLES

A workflow to identify novel proteins based on the direct mapping of Peptide-Spectrum-Matches to genomic locations

John Anders, Hannes Petruschke, Nico Jehmlich, Sven-Bastiaan Haange, Martin von Bergen and Peter F Stadler

Supplemental Figures

Supplemental Figure 1. The number of proteins that are identified only with the `proteome` and the `6frame` databases for the *E. coli* data set, respectively, as a function of the minimal number k of PSMs required to call a protein. The graph summarizes the stacked bar plot corresponding to Fig. 1 of the the main text.

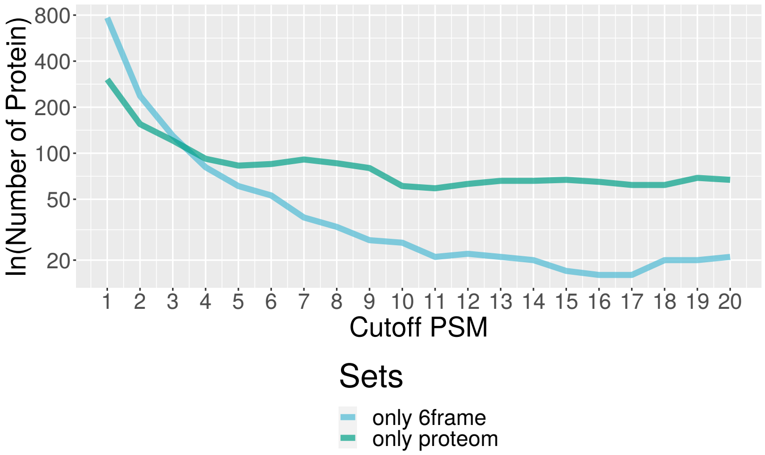

Supplemental Figure 2. False discovery rate (FDR) of proteins as a function of the minimum number of PSMs required to call a protein candidate of *E. coli*. The FDR_{prot} is estimated from the number of non-annotated candidates, assuming that the *E. coli* annotation is complete.

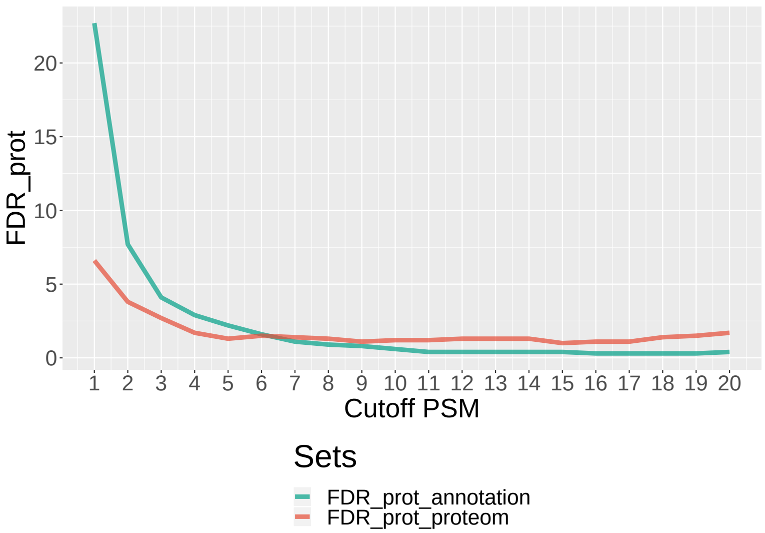

Supplemental Figure 3. The relation of the genomic (red) and annotation (blue) based $FDR_{genome/anno}$ and the FDR_{decoy} inferred by the target decoy method. The black line represents a perfectly proportional relationship between $FDR_{genome/anno}$ and FDR_{decoy}. The $FDRs$ are plotted for all experimental replicas.

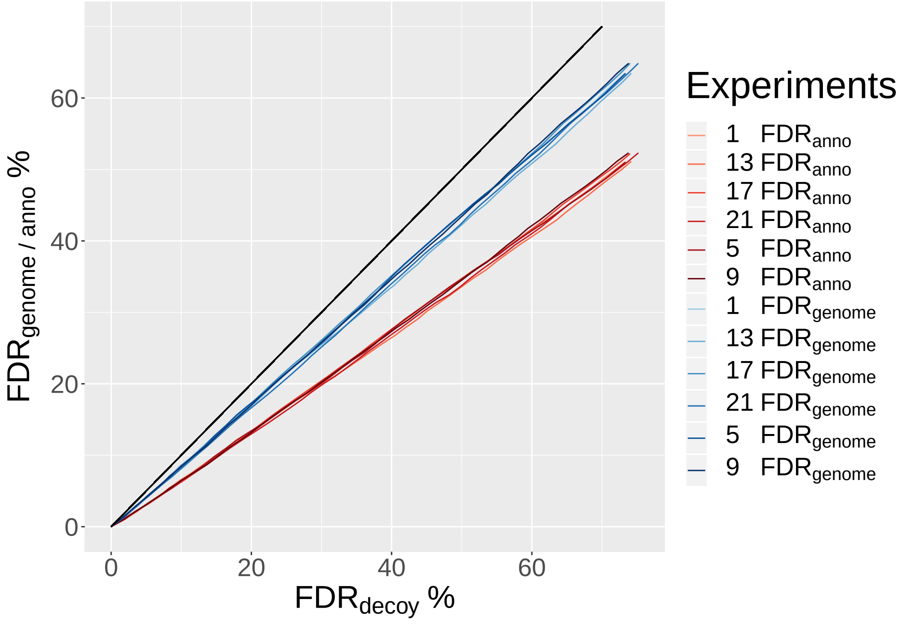

Supplemental Figure 4. Protein candidate 57 in *B. producta*, for which only hypothetical homologs can be found. The top of the figure shows a view in the UCSC Genome Browser. The first track show the reading frame of the genome on the negative strand. The second track is the protein candidate 5's predicted ORF. The next track shows a bar plot representation the reads per base mapping to protein candidate 5 from the transcriptomic data. At the bottom a list of the mapped PSMs can be found. The rightmost PSM is ambiguous and provides little evidence for the candidate. Below three mass spectra for top confidence PSMs and the corresponding peptides are shown.

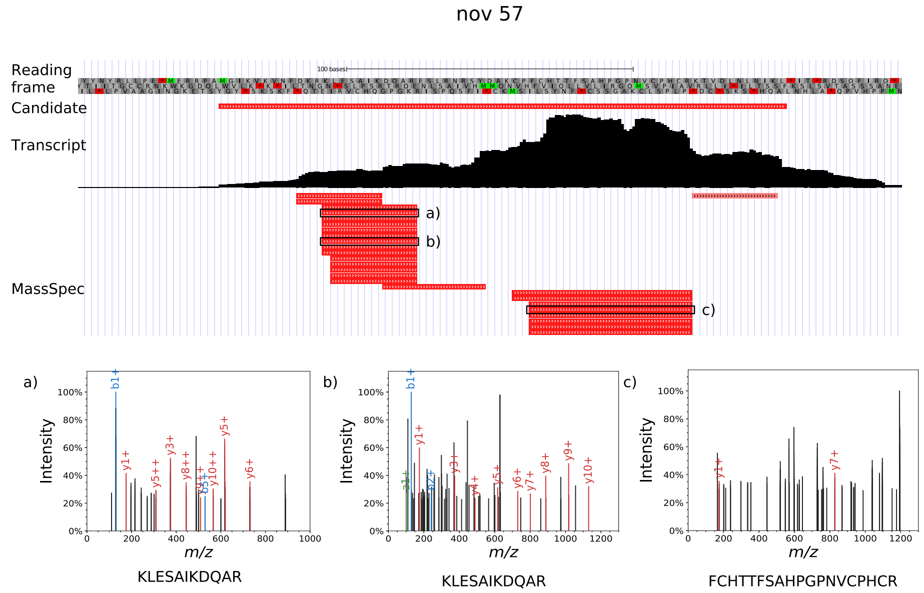

Supplemental Tables

Supplemental Table 1. Comparison of predicted protein candidates between `6-frame` database and a proteome database based on existing annotation including hypothetical protein.

	10 PSM						
	nov	hypo		known		$\sum$	
species	`6-frame`	proteome	`6-frame`	proteome	`6-frame`	proteome	`6-frame`
B. theta.	37	1975	2079	248	254	2500	4706
B. producta	52	1138	1214	132	138	1559	2857
E. coli	26	150	136	988	1061	1370	2538
E. ramosum	10	355	373	53	62	541	944
B. longum	16	128	153	0	0	202	356
A. caccae	17	549	597	100	103	841	1456
L. plantarum	31	83	109	28	39	194	342
C. butyricum	14	135	181	32	37	303	422

	6 PSM						
	nov	hypo		known		$\sum$	
species	`6-frame`	proteome	`6-frame`	proteome	`6-frame`	proteome	`6-frame`
B. theta.	72	2118	2238	256	262	2500	4706
B. producta	103	1289	1411	143	148	1559	2857
E. coli	65	182	183	1127	1187	1370	2538
E. ramosum	30	431	465	65	76	541	944
B. longum	42	170	202	0	0	202	356
A. caccae	39	632	721	119	120	841	1456
L. plantarum	48	116	147	36	47	194	342
C. butyricum	26	176	257	39	46	303	422

Supplemental Table 2. Full list of species in SIHUMIx and the corresponding NCBI assembly and taxonomy ID.

full name	taxid	assembly id
Anaerostipes caccae DSM 14662	411490	GCA_014131675.1
Bacteroides thetaiotaomicron VPI5482	226186	GCA_014131755.1
Bifidobacterium longum NCC2705	206672	GCF_000007525.1
Blautia producta ATCC 27340 DSM 2950	1121114	GCA_014131715.1
Clostridium butyricum DSM 10702	1316931	GCA_014131795.1
Escherichia coli str K12 substr MG1655	511145	GCF_000005845.2
Erysipelatoclostridium ramosum DSM 1402	445974	GCA_014131695.1
Lactobacillus plantarum subsp plantarum ATCC 14917 JCM 1149 CGMCC 12437	525338	GCA_014131735.1
